# Ginkgolic Acid Inhibits VSMC Proliferation and Migration and Vascular Restenosis by Regulating Cell Cycle Progression and Cytoskeleton Rearrangement Through TCTN1

**DOI:** 10.3390/cells14231922

**Published:** 2025-12-03

**Authors:** Yuting Shao, Lingyan Yi, Qingyu Zhu, Yulin Zhou, Tingting Chen, Wenjuan Yao

**Affiliations:** School of Pharmacy, Nantong University, 9 Seyuan Road, Nantong 226019, China; 2323310025@stmail.ntu.edu.cn (Y.S.); 2423310027@stmail.ntu.edu.cn (L.Y.); 1923110158@stmail.ntu.edu.cn (Q.Z.); 2023110118@stmail.ntu.edu.cn (Y.Z.); 2234110094@stmail.ntu.edu.cn (T.C.)

**Keywords:** Ginkgolic acid, vascular restenosis, vascular smooth muscle cell, tectonic family member 1, CCCTC binding factor

## Abstract

Ginkgolic acid (GA) exhibits various biological activities, but its role in vascular restenosis remains unreported. GA (13:0) is a relatively abundant natural congener. This study aims to investigate and clarify the effects and mechanisms of GA (13:0) on vascular smooth muscle cell (VSMC) proliferation and migration in vitro, as well as on balloon injury-induced vascular restenosis in rats. The results showed that GA (13:0) significantly inhibited VSMC proliferation, migration, and intimal thickening both in vitro and in vivo. Moreover, GA (13:0) reduced the expression of cyclin D1, cyclin E1, CDK2, and CDK4, as well as cyclin D1-CDK4 and cyclin E1-CDK2 binding, leading to G0/G1 arrest. Additionally, GA (13:0) suppressed vimentin expression and actin cytoskeleton polymerization and altered F-actin morphology. Comparative proteomics identified tectonic family member 1 (TCTN1) as a potential molecular target of GA (13:0). GA (13:0) reduced TCTN1 expression both in vitro and in vivo. Crucially, TCTN1 overexpression notably reversed the inhibitory effects of GA (13:0) on VSMC proliferation, migration, intimal thickening, expression and binding of cell cycle-related proteins, and vimentin expression. Concurrently, TCTN1 overexpression also reversed GA (13:0)-induced F-actin depolymerization and rearrangement and G0/G1 arrest. GA (13:0) significantly inhibited TCTN1 co-localization with vimentin and actin in vitro and in vivo. Furthermore, we found that CCCTC binding factor (CTCF) binds to the 162–176 site of the TCTN1 promoter to regulate TCTN1 transcription, and CTCF knockout significantly down-regulated TCTN1 protein levels. This study reveals that GA (13:0) inhibits TCTN1 transcription and expression, hindering G1/S transition, vimentin expression, and F-actin rearrangement, thereby suppressing vascular restenosis.

## 1. Introduction

Vascular restenosis, a common cardiovascular pathology [1], exhibits progressive characteristics. Factors such as atherosclerotic plaque progression and thrombus formation following vascular endothelial injury lead to progressive vessel lumen stenosis, triggering hemodynamic disorders, insufficient tissue perfusion, and severe clinical complications. Studies have shown that vascular neointima formation is a key contributor to restenosis. The change in the phenotype of vascular smooth muscle cells (VSMCs) from a contractile to a synthetic phenotype is fundamental to the process of neointima formation [2,3,4]. VSMCs, the primary cells in the vascular tunica media, are essential for maintaining vascular tone and hemodynamic homeostasis [5]. In their mature state under normal physiological conditions, these cells display a differentiated/contractile phenotype, characterized by elevated levels of contractile markers like α-smooth muscle actin (α-SMA) and smooth muscle 22α (SM22α) [6]. However, vascular injury or pathological stimuli can induce VSMC phenotypic transformation to a dedifferentiated/synthetic phenotype, characterized by enhanced proliferation and migration, extracellular matrix secretion, and neointima formation, resulting in vascular lumen stenosis [7]. Such an imbalance in phenotypic adaptability serves as a central pathological mechanism in various cardiovascular disorders, including atherosclerosis, vascular restenosis, and hypertension [8]. Therefore, targeted regulation of VSMC phenotypic transformation is a vital strategy for preventing and treating cardiovascular diseases.

Current drugs targeting VSMC phenotypic transformation include statins, paclitaxel, mTOR inhibitors, and tacrolimus [9,10]. Nonetheless, there has been no clinical trial that has investigated the local administration of statins for the prevention of restenosis [9]. Paclitaxel, an anti-tumor drug, significantly damages vascular endothelium and increases mortality risk [9,11]. Tacrolimus has significant nephrotoxicity and neurotoxicity [12,13]. Although mTOR inhibitors are preferred in coronary interventions, they cause side effects like poor tissue retention, immunosuppression, and metabolic abnormalities [14]. Recently, natural phytocompounds have emerged as promising candidates for managing neointimal hyperplasia. Polyphenols such as resveratrol and quercetin, which occur naturally, suppress the activity of the mitogen-activated protein kinase (MAPK), nuclear factor kappa B (NF-κB), and Notch pathways. This suppression inhibits VSMC proliferation and migration, helping to prevent both neointimal hyperplasia and vascular restenosis [15,16]. The resveratrol analog (R)-TML104 also exerts similar effects by upregulating SIRT1, reducing NF-κB acetylation, downregulating nicotinamide adenine dinucleotide phosphate oxidase 4 (NOX4) expression, and inhibiting platelet-derived growth factor (PDGF)-BB-induced VSMC phenotypic transformation, thereby alleviating neointima formation [17]. Curcumin inhibits neointimal hyperplasia by blocking key signaling pathways such as phosphoinositide 3-kinase (PI3K)/Akt, extracellular signal-regulated kinase (ERK) 1/2, and NF-κB, reducing VSMC proliferation, migration, and extracellular matrix synthesis [18,19,20].

*Ginkgo biloba* (*G. biloba*) extract (GBE) has broad applications in treating cardiovascular diseases [21]. Studies have shown that GBE significantly inhibits VSMC proliferation in vitro and intimal hyperplasia and arterial restenosis in vivo [22,23]. Ginkgolic acids (GAs), the main phenolic components in *G. biloba* leaves, comprise six different structures: C13:0, C15:0, C15:1, C17:1, C17:2, and the unknown C17:3 [24]. Although GAs contribute to the toxicity or allergic reactions associated with *G. biloba* seeds, their potent insecticidal, bactericidal [25], anti-tumor [26], anti-viral [27], anti-inflammatory [28], and antioxidant properties have garnered significant attention. In addition, GAs act as SUMOylation inhibitors [29], holding important potential in cancer therapy. GAs significantly reduce the synthesis of nitric oxide (NO), prostaglandin E2 (PGE2), and pro-inflammatory cytokines in human umbilical vein endothelial cells (HUVECs), reducing endothelial inflammation and improving intima function [30]. Nevertheless, there has been no reported evidence regarding the ability of GAs to specifically target and inhibit VSMC phenotypic transformation and vascular restenosis.

Based on these considerations, the objective of this research was to assess the impacts of GA (13:0) on the phenotypic transformation of VSMCs induced by PDGF-BB in vitro, as well as on intimal thickening and restenosis induced by balloon injury (BI) in vivo. Furthermore, we evaluated the role of the target tectonic family member 1 (TCTN1) in this process.

## 2. Materials and Methods

### 2.1. Materials

Ginkgolic acid C13:0 (GA 13:0) (CAS No.: 20261-38-5) was bought from Chenguang Biotechnology Co., Ltd. (Baoji, China). PDGF-BB (Cat. No. GMP-C199) was bought from Jinan Protein Technology Co., Ltd. (Suzhou, China). Antibodies against cyclin-dependent kinase 2 (CDK2) (AF1063), CDK4 (AF2515), cyclin D1 (AF1183), cyclin E1 (AF2494), vimentin (AF0318), actin (AA128), CTCF (AF6579), and rabbit IgG (A7016) were purchased from Beyotime Biotechnology Co., Ltd. (Shanghai, China). Polyclonal antibodies against TCTN1 (15004-1-AP), GAPDH (60004-1), Cy3-conjugated IgG (H+L) (SA00009–1), and Fluorescein (FITC)-conjugated IgG (H+L) (SA00003-8) were bought from Proteintech (Chicago, IL, USA). Antibodies against CD31 (AFRM0001), α-SMA (AFRM0018), and ki67 (AFRP0030) were bought from AiFang Biotechnology (Changsha, China). Antibodies against β-tubulin (AT0003) and CTCF (D31H2) XP^®^ (#3418) were purchased from CMCTAG Inc. (San Diego, CA, USA) and Cell Signaling Technology (Beverly, MA, USA), respectively. The CellLight™EdU Apollo^®^ 567 In vitro Imaging Kit (C10310-1) was purchased from RiboBio Co., Ltd. (Guangzhou, China). Transwell systems (8.0 mm pore size) (#3422) were purchased from Corning, Inc. (Corning, NY, USA). The crystal violet staining solution (C0121), SDS-PAGE gel rapid preparation kit (P0012A), RIPA lysis buffer (strong) (P0013B), Cell Cycle and Apoptosis Detection Kit (C1052), PMSF (100 mM) (ST506), DAPI staining solution, and BeyoFast™ SYBR Green qPCR Mix (2×, High ROX) (D7265) were purchased from Beyotime Biotechnology Co., Ltd. (Shanghai, China). Fogarty 2 F balloon catheter (#12TLW804F) was bought from Baxter Healthcare Corp. (Irvine, CA, USA). MMC (mitomycin C) (#YZ-1444707) and 10 × TBST (#T1081) were bought from Solarbio (Shanghai, China). The universal tissue fixative (neutral) (G1101), dewaxing liquid (G1128), Hematoxylin-Eosin (H&E) staining kit (G1076), Masson stain kit (G1006), bovine serum albumin (BSA) (GC305010), and histochemistry kit DAB chromogenic agent (G1212) were bought from Servicebio Technology Co., Ltd. (Wuhan, China). Protein A/G magnetic beads (HY-K0202) were purchased from MedChemExpress (Shanghai, China). The G-actin/F-actin In vivo Assay Kit (BK037) was purchased from Cytoskeleton, Inc. (Denver, CO, USA). iFluor™ 488-labeled phalloidin (green) (#40736ES75) was bought from Yeasen Biotechnology (Shanghai, China). Total RNA Purification Kit was from TIANGEN Biotech (Beijing, China). The RevertAid First Strand cDNA Synthesis Kit (#K1622) and Dream Taq PCR Master Mix (#K1071) were purchased from Thermo Scientific (Shanghai, China). Other chemicals used in this study were of analytical grade and were made in China.

### 2.2. Cell Culture and Treatment

Human aortic VSMCs (HA-VSMCs) were bought from the American Type Culture Collection (ATCC number: CRL-1999; Manassas, VA, USA) and cultured as we previously described [3]. Cells in passages 3–7 were used in this study. The recombinant plasmid pcDNA3.1-EGFP-TCTN1 with overexpression of TCTN1 was synthesized by Gene Create Biology (Wuhan, China). The plasmid pcDNA3.1-EGFP was used as a negative control. Cells were transfected with 2.5 μg plasmid DNA by Lipofectamine™ 2000 for 48 h (Thermo Fisher Scientific™, Waltham, MA, USA). On the day of transfection, the cells were pretreated with various concentrations of GA (13:0) (0.2, 0.4, 0.8, 1 μM) for 24 h, followed by an additional treatment with 10 ng/mL PDGF-BB for another 24 h.

### 2.3. EdU Assay

An equal amount of the pre-heated 2 × EdU working solution (20 μM) was introduced to the cells, followed by a 2 h incubation period. After that, 1 mL of fixative was applied to the cells for 15 min, after which they were rinsed 3 times with the washing solution. Subsequently, 1 mL of permeabilization solution was added to cells and left at room temperature for 10–15 min. In the final step, cells were treated with 0.5 mL of click reaction solution in the dark at room temperature for 30 min and then subjected to Hoechst 33342 staining. Images were obtained using a fluorescence microscope (Olympus, Tokyo, Japan)

### 2.4. Cell Migration Assay

Cell migration was assessed through the wound healing and transwell assays. For detailed procedures, please see our earlier publication [3]. The number of cells that migrated from the scratched area or the cells stained with crystal violet were quantified and averaged based on the images obtained.

### 2.5. Rat Carotid Artery BI Model

Male Sprague-Dawley (SD) rats, aged between 40 and 45 days and weighing around 250 g, were sourced from the Animal Center at Nantong University (Nantong, China). The animal procedures adhered to the standards set by the Ethics Committee and the Animal Care and Use Committee of Nantong University (with Ethics Committee Approval No. 1213201.1), as well as the NIH Guidelines for Laboratory Animal Care and Use. The rats were kept in a temperature-controlled, pathogen-free environment (maintained at 22 ± 1 °C) and were provided with unrestricted access to food and water, following a 12 h light and dark cycle. Forty-eight SD rats were randomly assigned (*n* = 8/group; 4 mice in a cage) into the sham-operation, BI model, BI + 10 mg/kg GA (13:0), BI + 20 mg/kg GA (13:0), BI + 40 mg/kg GA (13:0), and BI + 40 mg/kg GA (13:0) + Lenti-TCTN1-treated groups [21,22,23,31]. Different doses of GA (13:0) were administered by intraperitoneal injection every day. The lentiviral vectors specific for TCTN1 overexpression were purchased from Azenta Life Sciences (Beijing, China). The BI model for the rat carotid artery and the lentiviral treatments were conducted as previously described [31,32,33]. After a period of fourteen days, rats were anesthetized with a mixture of ketamine (100 mg/kg) and xylazine (5 mg/kg) prior to euthanasia to minimize discomfort and stress. Humane euthanasia of the rats was carried out using CO_2_ at an infusion rate ranging from 10% to 30% per minute. Subsequently, the injured portions of the carotid artery, along with liver and kidney tissues, were isolated and rinsed with chilled normal saline.

### 2.6. H&E Staining and Masson’s Trichrome Staining

H&E staining and Masson’s trichrome staining were performed as previously described [31].

### 2.7. Immunohistochemical Analysis

Immunohistochemical analyses were conducted following previously established protocols [31].

### 2.8. Immunofluorescence Staining

Immunofluorescence staining was conducted following previously established protocols [31]. Immunofluorescence intensity and colocalization were, respectively, quantified by calculating the fluorescence intensity per μm^2^ and Pearson correlation coefficient (R value) using ImageJ 2.0 plugin Coloc 2.

### 2.9. Western Blotting

Western blotting was conducted following previously established protocols [31].

### 2.10. Quantitative Real-Time Polymerase Chain Reaction (qRT-PCR)

qRT-PCR was conducted following previously established protocols [31]. Specific primers for PCR (Table 1) were obtained from Biomics Biotechnologies (Nantong, China). The amplification conditions used for PCR cycling were as follows: 94 °C for 30 s, 54 °C for 35 s, 72 °C for 30 s, 30 cycles (vimentin); 94 °C for 30 s, 56 °C for 35 s, 72 °C for 35 s, 30 cycles (TCTN1); 94 °C for 30 s, 52 °C for 35 s, 72 °C for 20 s, 30 cycles (GAPDH). To determine the relative expression levels of vimentin and TCTN1, the 2^−ΔΔCt^ methodology was utilized.

### 2.11. Cell Cycle Analysis

The analysis of the cell cycle was conducted utilizing the Cell Cycle and Apoptosis Detection Kit alongside flow cytometry techniques. In summary, cells that had been treated were fixed using 70% ethanol overnight before being rinsed with PBS. Afterward, the cells underwent incubation with 100 μL of RNase A at 37 °C for 30 min, followed by staining with 400 μL of PI in the dark at 4 °C for an additional 30 min. A Beckman Coulter CytoFLEX (Pasadena, CA, USA) was employed to carry out the cell cycle analysis.

### 2.12. Co-Immunoprecipitation (Co-IP)

Treated cells were lysed with lysis buffer containing PMSF (1 mM) for 40 min on ice. The lysates were then centrifuged for 15 min and the supernatant was collected to determine the protein concentration. The protein samples were incubated with specific antibodies or IgG (negative control) overnight at 4 °C, and then incubated with Protein A/G magnetic beads at 4 °C for 2 h. Subsequently, the mixture was washed several times with washing buffer to remove unbound proteins. Pulled down proteins were added to 1 × SDS loading buffer and boiled at 95–100 °C for 5 min. The eluted protein samples were analyzed by Western blot.

### 2.13. F-Actin/G-Actin Ratio Assay

Cells underwent incubation with 1500 μL of warm LAS2 and were harvested by using a cell scraper. The resulting lysates were kept at 37 °C for 10 min before being centrifuged at 350× *g* for 5 min. Following this, the supernatants underwent additional centrifugation at 100,000× *g* for 1 h to pellet F-actin. Subsequently, the supernatant fractions containing G-actin were transferred into new tubes. The pellets containing F-actin were then incubated with 1500 μL of F-actin depolymerization buffer for 1 h. Finally, both the pellet and supernatant fractions were subjected to Western blot analysis for the quantitation of actin.

### 2.14. F-Actin Morphology

Cells underwent fixation using 4% paraformaldehyde for a duration of 20 min and subsequently permeabilization with 0.2% Triton X-100 for 30 min. Following this, the cells were stained with phalloidin labeled with iFluor™ 488 for 30 min, along with DAPI for an additional 15 min. Visualization of the stained cells was accomplished using a confocal laser scanning microscope (FV500; Olympus, Tokyo, Japan). The analysis of F-actin fiber directionality, dispersion, total area, and density was performed utilizing Image-Pro Plus 6.0 software (Media Cybernetics, Rockville, MD, USA).

### 2.15. Screening of Molecular Targets of GA (13:0)

The potential molecular targets of GA (13:0) were examined using iTRAQ (Isobaric Tag for Relative Absolute Quantitation) and TMT (Tandem Mass Tags). The analysis involved cells from both the PDGF-BB-treated group and the group treated with 1 μM GA (13:0) in conjunction with PDGF-BB. The mass spectrometry (MS) data underwent identification and quantitation analysis through the MASCOT engine (Matrix Science, London, UK; version 2.2). The top 20 up- and down-regulated proteins were used for clustering heatmap analysis. For further details, please refer to our previously published report [34].

### 2.16. Identification of Transcription Factors and Knockout of CTCF

The transcription factors were predicted through TF-Target Finder (https://jingle.shinyapps.io/TF_Target_Finder/ (accessed on 12 September 2024)). The human CCCTC binding factor (CTCF) gene was knocked out by CRISPR/Cas9 editing technology completed in Cyagen Company. The sgRNA sequences were as follows: CTCF-G1: TACACTGGCGTAATCGCACA; CTCF-G2: ATGCCGAACCAATTCTCCAC; CTCF-G3: AACACTTGAATGGCTTCTCG; CTCF-G4: ACACTTGAATGGCTTCTCGT. The result of CTCF knockout was verified by Western blot.

### 2.17. ChIP-qPCR

CTCF and TCTN1 promoter binding sites were predicted through JASPAR (https://jaspar.elixir.no/ (accessed on 6 November 2024)). Cells were re-suspended in 1 mL of ChIP lysis buffer and then subjected to sonication (working time: 30 s, interval time: 30 s, ultrasonic power: 99%, total time: 40 min). Part of the lysates was used as the input positive control, and the other part was incubated with the CTCF antibody or IgG (negative control) at 4 °C overnight with rotation. The lysate-CTCF antibody complex was incubated with Protein G magnetic beads at 4 °C for 4 h with rotation. Non-specific binding was washed with ChIP wash buffer. The target DNA was eluted with ChIP elution buffer. The supernatant containing DNA was digested with proteinase K and then micro-purified. The purified DNA was analyzed by qPCR. The specific primers for different binding sites were shown in Table 2. The amplification condition used for PCR cycling was as follows: 95 °C for 30 s, 95 °C for 5 s, 60 °C for 30 s, 40 cycles.

### 2.18. Dual-Luciferase Reporter Assay

The TCTN1 promoter binding sites that were predicted earlier were inserted into the pGL3-basic vector. To confirm these plasmids, sequencing was conducted. Subsequently, the recombinant plasmids along with the pRL-TK vector were co-transfected into HEK293T cells. A dual luciferase reporter assay was carried out and the activities of firefly luciferase (FFL) were normalized to those of Renilla (RL) luciferase.

### 2.19. Statistical Analyses

All of the results are expressed as the mean ± SD. One-way ANOVA followed by Tukey’s post hoc testing in SPSS V22.0 (IBM, New York, NY, USA) was used for the statistical analysis. Differences with a value of *p* < 0.05 were considered to be statistically significant.

## 3. Results

### 3.1. GA (13:0) Inhibits HA-VSMC Proliferation and Migration In Vitro and Neointima Formation In Vivo

**Figure 1:**
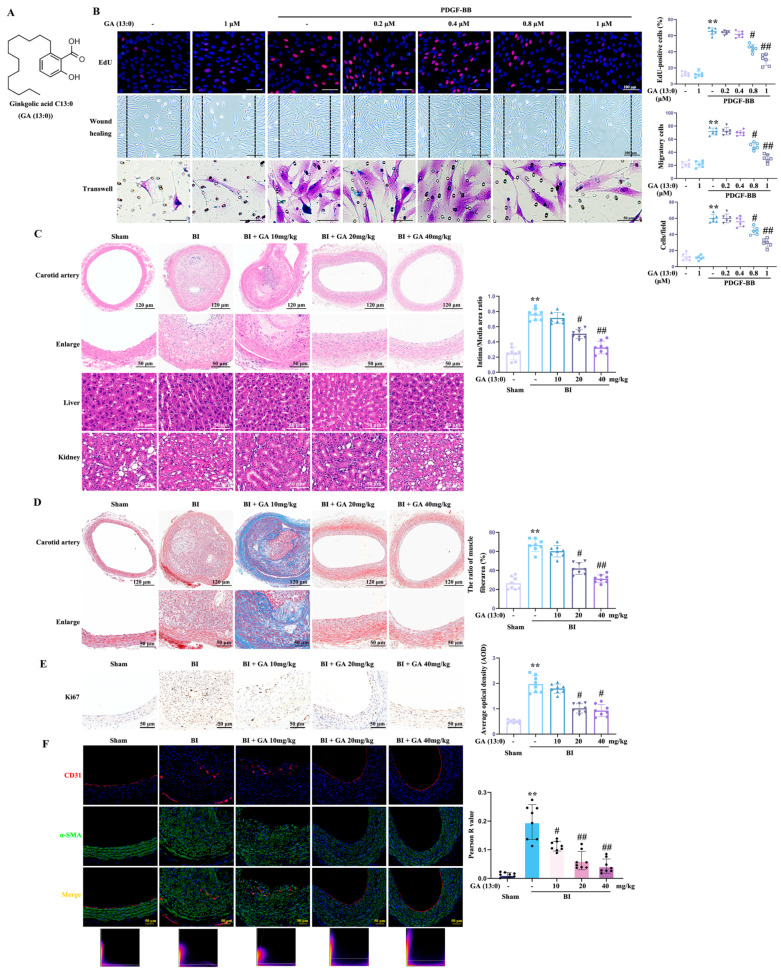
GA (13:0) significantly reduces PDGF-BB-induced HA-VSMC proliferation and migration in vitro, as well as balloon-injured neointima formation in vivo. HA-VSMCs were pretreated with different concentrations of GA (13:0) (0.2, 0.4, 0.8, 1 μM) for 24 h, and then treated with 10 ng/mL PDGF-BB for another 24 h. Rats underwent carotid artery BI and i.p. injection of GA (13:0) (10, 20, 40 mg/kg) once a day. (**A**) Chemical structure of GA (13:0). (**B**) The impacts of GA (13:0) on cell proliferation and migration induced by PDGF-BB. Proliferation of cells was evaluated through the EdU assay. Migration was measured using both wound-healing and transwell assays. Histograms illustrate the ratio of EdU-positive cells (red), the number of cells migrating from scratched boundary, and the cells stained with crystal violet that moved to the lower chamber. **, *p* < 0.01 compared to the control group; #, *p* < 0.05 and ##, *p* < 0.01 compared to the PDGF-BB-treated group (*n* = 6). (**C**) Typical images of H&E staining for carotid arteries, liver, and kidney 14 days post-BI. Histogram displays the area ratio of intima to media. **, *p* < 0.01 in comparison to the sham group; #, *p* < 0.05 and ##, *p* < 0.01 in relation to the BI group (*n* = 8). (**D**) Typical images of Masson’s trichrome staining for carotid arteries. Histogram represents the percentage of smooth muscle fiber area (%). **, *p* < 0.01 relative to the sham group; #, *p* < 0.05 and ##, *p* < 0.01 when compared to the BI group (*n* = 8). (**E**) Immunohistochemistry of ki67. Histogram indicates the average optical density (AOD) of ki67. **, *p* < 0.01 vs. the sham group; #, *p* < 0.05 vs. the BI group (*n* = 8). (**F**) Immunofluorescence staining showing the colocalization of CD31 (red) with α-SMA (green). DAPI (blue) was used to stain the nuclei. The lower panel presents the Pearson R values for the corresponding images. Histogram illustrates the Pearson R value of α-SMA-CD31 colocalization. **, *p* < 0.01 compared to the sham group; #, *p* < 0.05 and ##, *p* < 0.01 compared to the BI group (*n* = 8).

While GA (15:1) regulates C2C12 myoblast proliferation and differentiation [35], its effects on VSMC phenotypic transformation and vascular restenosis remain ambiguous. This research aimed to examine how GA (13:0) (Figure 1A) affects the proliferation and migration of PDGF-BB-stimulated HA-VSMCs and the vascular intimal thickening mediated by BI. In vitro, EdU, wound healing, and transwell assays were utilized to evaluate the effects of different concentrations of GA (13:0) on HA-VSMC proliferation and migration. As illustrated in Figure 1B, pretreatment with 0.8 and 1 μM GA (13:0) notably reduced PDGF-BB-mediated cell proliferation and migration. In contrast, lower concentrations of GA (13:0) at 0.2 and 0.4 μM showed minimal effects. Compared with the control group, treatment with 1 μM GA (13:0) alone had no significant effect on HA-VSMC proliferation and migration (Figure 1B).

In the rat carotid artery restenosis model mediated by BI in vivo, HE staining showed that intraperitoneal (i.p.) injection of 20 and 40 mg/kg GA (13:0) significantly reduced intima/media area ratios of carotid arteries (0.76 ± 0.11 vs. 0.51 ± 0.08, *p* < 0.05; 0.76 ± 0.11 vs. 0.33 ± 0.09, *p* < 0.01) when compared with the BI group (Figure 1C). However, 10 mg/kg GA (13:0) had no significant effect on BI-mediated vascular intimal thickening. Given reports of GAs-related liver and kidney toxicity [24], this study evaluated the impact of different GA (13:0) doses (10, 20, and 40 mg/kg, i.p.) on rat liver and kidney structures using HE staining. As shown in Figure 1C, GA (13:0) administration at all doses had no significant effect on liver or kidney structure compared to the sham group. Hepatocytes and renal cells remained orderly arranged, with uniform nuclear sizes and no observable inflammatory cell infiltration. Masson staining (Figure 1D) demonstrated that i.p. injection of 20 mg/kg and 40 mg/kg GA (13:0) dramatically inhibited the increase in the proportion of smooth muscle fiber area induced by BI (66.6 ± 7.4 vs. 42.0 ± 7.8, *p* < 0.05; 66.6 ± 7.4 vs. 31.1 ± 6.9, *p* < 0.01). The 10 mg/kg dose had no significant effect. In order to further assess whether GA (13:0) inhibits the proliferation and migration of rat VSMCs in vivo, we performed immunostaining for ki-67 expression and examined the colocalization of CD31 (an endothelial marker) with α-SMA (a myofiber marker). As shown in Figure 1E, i.p. administration of 20 mg/kg and 40 mg/kg GA (13:0) significantly attenuated the BI-induced increase in the average optical density (AOD) of ki-67 and markedly reduced the number of ki-67-positive cells. Treatment with 10 mg/kg GA (13:0) did not affect BI-mediated ki-67 expression. As illustrated in Figure 1F, i.p. injection of 10, 20, and 40 mg/kg GA (13:0) significantly reduced both the Pearson R value and the extent of CD31-α-SMA co-localization induced by BI. These results indicate that GA (13:0) significantly inhibits the proliferation and migration of rat VSMCs following BI, consistent with the in vitro results.

### 3.2. GA (13:0) Regulates VSMC Proliferation and Migration by Modulating Cyclins and Cytoskeletal Proteins

Cell division and proliferation are closely linked to cell cycle progression. Numerous cell cycle regulators controlling DNA replication, mitosis, and arrest, such as cyclins and cyclin-dependent kinases (CDKs), have been identified [36]. Specifically, the G1/S transition is regulated by the activities of cyclin D:CDK4 in early G1 and cyclin E:CDK2 in late G1 [37]. To investigate the impact of GA (13:0) on cell cycle progression, this study assessed the expression and binding of key regulators via Western blot and co-immunoprecipitation, respectively, and analyzed cell cycle distribution using flow cytometry.

**Figure 2:**
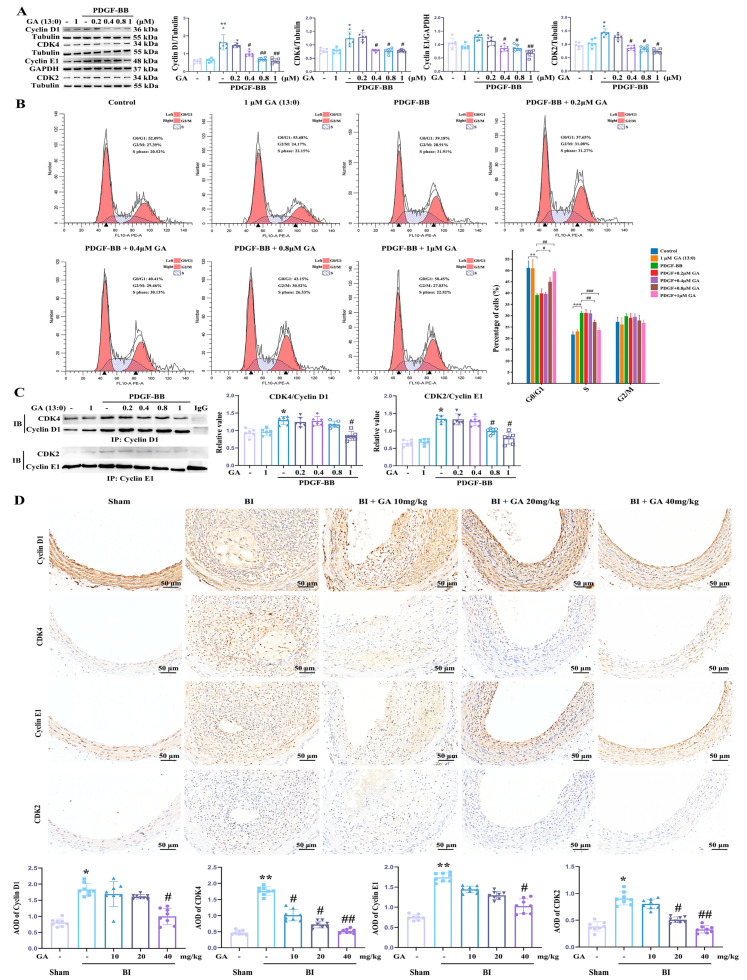
GA (13:0) induces an arrest in the G0/G1 phase by influencing proteins associated with the cell cycle. The treatment methods for cells and rats are the same as those in Figure 1. (**A**) Western blotting was utilized to analyze cyclin D1, CDK4, cyclin E1, and CDK2 levels. The accompanying histograms depict the ratios of cyclin D1, CDK4, CDK2 to tubulin or cyclin E1 to GAPDH. *, *p* < 0.05 and **, *p* < 0.01 when compared to the control group; #, *p* < 0.05 and ##, *p* < 0.01 relative to the PDGF-BB-treated group (*n* = 6). (**B**) The progression through the cell cycle was assessed through flow cytometry, employing propidium iodide (PI) staining. The histogram represents quantification of the cell cycle across various groups. **, *p* < 0.01 and ***, *p* < 0.001 compared to the control group; #, *p* < 0.05, ##, *p* < 0.01 and ###, *p* < 0.001 vs. the PDGF-BB-treated group. (**C**) Co-immunoprecipitation (Co-IP) was conducted to analyze the interaction between cyclin D1 and CDK4 or cyclin E1 and CDK2. Specific antibodies were used to immunoprecipitate cyclin D1 and cyclin E1 from the cell lysates. The precipitated proteins were then evaluated using Western blotting, with IgG serving as the negative control. The resulting histograms illustrate the ratios of CDK4 to cyclin D1 and CDK2 to cyclin E1. *, *p* < 0.05 vs. control group; #, *p* < 0.05 vs. PDGFBB-treated group (*n* = 6). (**D**) Immunohistochemical analysis of cyclin D1, CDK4, cyclin E1, and CDK2 was performed on carotid arteries in a rat BI model. Histograms present the average optical density (AOD) measurements of the target proteins. *, *p* < 0.05 and **, *p* < 0.01 in comparison to the sham group; and #, *p* < 0.05 and ##, *p* < 0.01 relative to the BI group (*n* = 8).

As shown in Figure 2A, the treatment with PDGF-BB notably enhanced the levels of cyclin D1, CDK4, cyclin E1, and CDK2 in comparison to the control group. Pretreatment with 0.4, 0.8, and 1 μM GA (13:0) markedly inhibited this PDGF-BB-induced elevation, whereas 0.2 μM GA (13:0) had no significant effect (Figure 2A). Treatment with 1 μM GA (13:0) alone did not significantly alter the expression of these regulatory proteins. The flow cytometry results (Figure 2B) revealed that PDGF-BB increased the proportion of cells in S phase from 21.60 ± 1.36% to 31.07 ± 0.84% and decreased the proportion in G0/G1 phase from 51.18 ± 2.45% to 39.07 ± 0.62%. Pretreatment with 0.8 and 1 μM GA (13:0) reduced the PDGF-BB-induced S phase population to 27.16 ± 0.98% and 23.59 ± 0.79%, respectively, and increased the G0/G1 phase population to 44.98 ± 1.77% and 49.64 ± 0.81%, respectively (Figure 2B). Pretreatment with 0.2 and 0.4 μM GA (13:0) had no significant effect on G0/G1 or S phase proportions in the PDGF-BB-induced model (Figure 2B). Neither PDGF-BB nor GA (13:0) at any concentration significantly affected the proportion of cells in G2/M phase (Figure 2B). Treatment with 1 μM GA (13:0) alone had no significant effect on cell cycle progression (Figure 2B). Additionally, as illustrated in Figure 2C, treatment with PDGF-BB led to a notable enhancement in the binding of cyclin D1 to CDK4 and cyclin E1 to CDK2. Pretreatment with 1 μM GA (13:0) significantly inhibited cyclin D1:CDK4 binding, while lower concentrations (0.2, 0.4, 0.8 μM) did not (Figure 2C). PDGF-BB-induced cyclin E1:CDK2 binding was dramatically suppressed by 0.8 and 1 μM GA (13:0), but unaffected by 0.2 and 0.4 μM (Figure 2C). Compared with the control group, 1 μM GA (13:0) alone did not affect the binding of cell cycle regulatory proteins. In vivo immunohistochemistry results showed that BI significantly increased the AOD values and the number of positive brown granules for cyclin D1, CDK4, cyclin E1, and CDK2 (Figure 2D). Intraperitoneal injection of 10 mg/kg GA (13:0) significantly inhibited the AOD value and the number of positive granules of CDK4, while 20 mg/kg GA (13:0) inhibited the number of positive granules of CDK2 and CDK4 (Figure 2D). Furthermore, GA (13:0) at 40 mg/kg simultaneously suppressed the expression of all four proteins.

**Figure 3:**
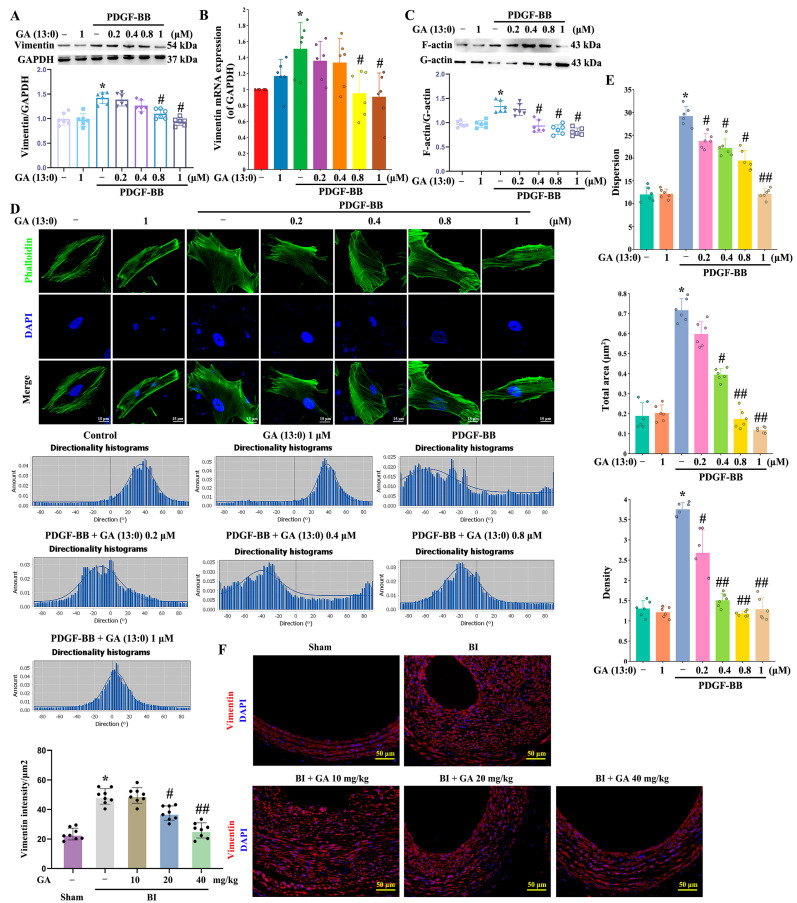
GA (13:0) regulates cell cytoskeleton by inhibiting vimentin expression and F-actin polymerization. The treatment methods for cells and rats are the same as those in Figure 1. (**A**) Vimentin was analyzed using Western blot. The histogram displays the vimentin to GAPDH ratio. *, *p* < 0.05 compared to the control group; #, *p* < 0.05 in comparison to the PDGF-BB-treated group (*n* = 6). (**B**) qRT-PCR analysis of vimentin transcription. Histogram shows the ratio of vimentin mRNA to GAPDH mRNA. *, *p* < 0.05 vs. the control group; #, *p* < 0.05 vs. the PDGF-BB-treated group (*n* = 6). (**C**) The ration of F-action to G-actin analyzed by G-actin/F-actin In vivo Assay Kit and Western blot analysis. Histogram shows the ratio of F-actin/G-actin. *, *p* < 0.05 vs. the control group; #, *p* < 0.05 vs. the PDGF-BB-treated group (*n* = 6). (**D**) F-actin filaments were stained by phalloidin (green). Nuclei were stained with DAPI (blue). The lower panels show F-actin fiber directionality histogram, showing the directions and the distribution ratios of each direction. (**E**) A quantitative analysis was performed to assess the dispersion, total area, and density of F-actin fibers in (**D**) using Image-Pro Plus software. *, *p* < 0.05 when compared to the control group; #, *p* < 0.05 and ##, *p* < 0.01 in relation to the PDGF-BB-treated group (*n* = 6). (**F**) immunofluorescence staining revealed vimentin (red), while the nuclei were visualized with DAPI (blue). The histogram presents vimentin intensity per μm^2^. *, *p* < 0.05 compared to the sham group; #, *p* < 0.05 and ##, *p* < 0.01 relative to the BI group (*n* = 8).

Our previous studies confirmed that vimentin cytoskeleton is involved in PDGF-BB-induced HA-VSMC migration [33]. It has been reported that actin reorganization participates in the migration of rat aortic VSMCs [38]. Therefore, in this study, we examined the effects of GA (13:0) on vimentin and actin cytoskeletons. As shown in Figure 3A,B, PDGF-BB significantly increased the transcription and expression of vimentin, which was inhibited by pretreatment with 0.8 and 1 μM GA (13:0). While 0.2 and 0.4 μM GA (13:0) had no significant impact on PDGF-BB-induced elevation of vimentin. Treatment with 1 μM GA (13:0) alone did not affect vimentin expression. The polymerization of globular actin (G-actin) into filamentous actin (F-actin) and the depolymerization of F-actin are key steps in regulating actin filament networks [39]. This study assessed the effect of GA (13:0) on actin dynamics by measuring the F-actin/G-actin protein ratio. As shown in Figure 3C, HA-VSMCs treated with PDGF-BB expressed dramatically higher levels of the F-actin/G-actin ratio, and this effect was inhibited by pretreatment with 0.4, 0.8, and 1 μM GA (13:0). 0.2 μM GA (13:0) pretreatment did not significantly affect PDGF-BB-induced increase in the F-actin/G-actin ratio (Figure 3C). 1 μM GA (13:0) treatment alone had no significant effect on the F-actin/G-actin ratio (Figure 3C). Furthermore, we observed the effect of GA (13:0) on the morphology of F-actin cytoskeleton using phalloidin staining (green). As shown in Figure 3D, PDGF-BB promoted the multi-directionality of F-actin fibers compared to the control. Pretreatment with 0.8 and 1 μM GA (13:0) significantly suppressed this multi-directional distribution, causing the F-actin fibers to align more unidirectionally. PDGF-BB also significantly promoted the dispersion, total area, and fiber density of F-actin (Figure 3E). GA (13:0) at 0.4, 0.8, and 1 μM significantly inhibited these PDGF-BB-induced effects (Figure 3E). GA (13:0) at 0.2 μM dramatically suppressed the increase in F-actin dispersion and fiber density induced by PDGF-BB but not the total area (Figure 3E). Compared with the normal group, treatment with 1 μM GA (13:0) alone did not significantly affect F-actin directional distribution, dispersion, total area, or fiber density (Figure 3D,E). The in vivo results, as shown in Figure 3F, demonstrated that BI significantly increased vimentin fluorescence intensity. This effect was inhibited by i.p. injection of 20 mg/kg and 40 mg/kg GA (13:0), but not by 10 mg/kg GA (13:0) (Figure 3F).

### 3.3. GA (13:0) Inhibits VSMC Proliferation, Migration and Neointima Formation by Suppressing TCTN1 Expression

**Figure 4:**
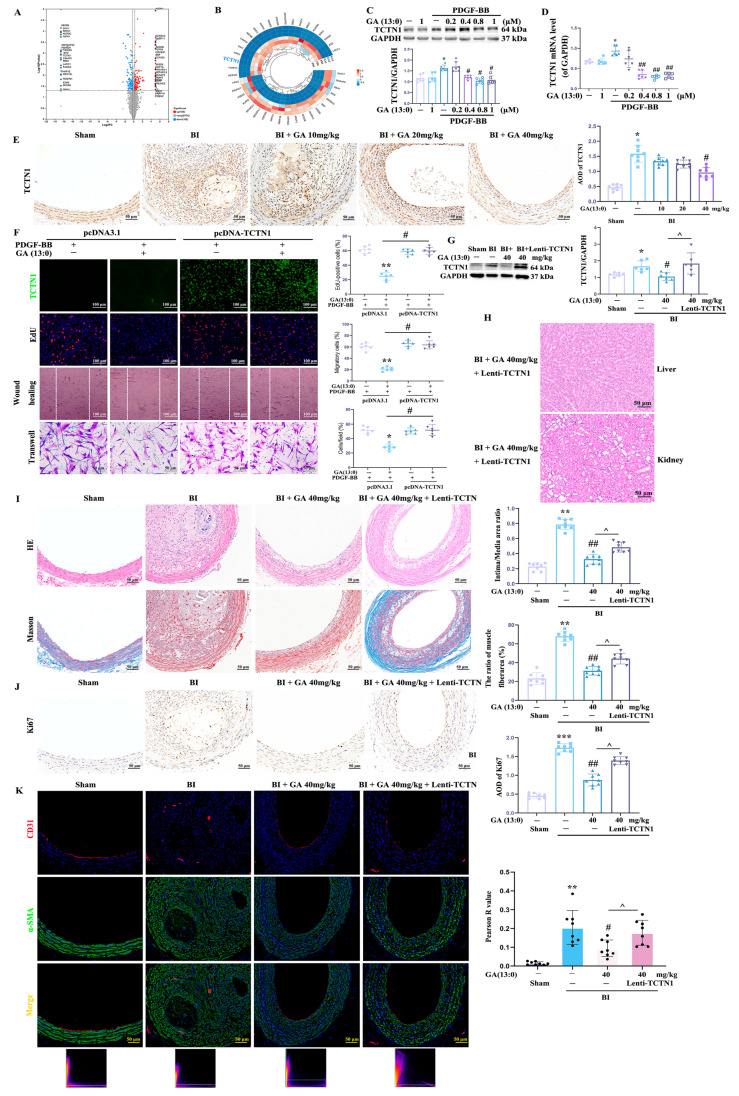
GA (13:0) reduces cell proliferation and migration and neointima formation by inhibiting TCTN1 expression. HA-VSMCs were transfected with the TCTN1-overexpression plasmid (pcDNA3.1-EGFP-TCTN1) or the control plasmid pcDNA3.1-EGFP. Then cells were treated with 1 μM GA (13:0) and 10 ng/mL PDGF-BB. Rats were treated with recombinant lentivirus for TCTN1 overexpression after BI and i.p. injected with GA (13:0) (40 mg/kg). (**A**) The volcano plot displayed the distribution and number of differentially expressed proteins (DEPs) between the PDGF-BB + 1 μM GA treatment group and the PDGF-BB group. A total of 108 proteins were identified as up-regulated, alongside 108 proteins that were down-regulated. Up-regulated proteins are represented by red dots, whereas blue dots correspond to down-regulated proteins. (**B**) A circular heatmap illustrating the top 20 DEPs from the PDGF-BB + 1 μM GA group was compared to the PDGF-BB group, with three biological replicates per group. The upregulation is marked in red, and downregulation is shown in blue. (**C**) A Western blot analysis was performed on TCTN1, with the histogram indicating the ratio of TCTN1 to GAPDH. *, *p* < 0.05 when compared to the control group; #, *p* < 0.05 compared to the PDGF-BB-treated group (*n* = 6). (**D**) qRT-PCR analysis was conducted to evaluate TCTN1 transcription levels, with the histogram depicting the ratio of TCTN1 mRNA to GAPDH mRNA. *, *p* < 0.05 against the control group; ##, *p* < 0.01 when compared to the PDGF-BB-treated group (*n* = 6). (**E**) For TCTN1, immunohistochemistry was performed, and the histogram illustrates the AOD of TCTN1. *, *p* < 0.05 vs. the sham group; #, *p* < 0.05 in comparison to the BI group (*n* = 8). (**F**) An analysis of TCTN1 overexpression was performed through immunofluorescence, alongside cell proliferation and migration assays. Histograms show the ratio of EdU-positive cells (red), migrating cells from scratched boundary, and crystal violet stained cells migrating to the lower chamber. *, *p* < 0.05 and **, *p* < 0.01 vs. the PDGF-BB + pcDNA3.1-EGFP group; #, *p* < 0.05 vs. the PDGF-BB + 1 μM GA + pcDNA3.1-EGFP group (*n* = 6). (**G**) Western blot analysis of TCTN1 from carotid arteries. Histogram shows the ratio of TCTN1 to GAPDH. *, *p* < 0.05 vs. the sham group; #, *p* < 0.05 vs. the BI group; ^, *p* < 0.05 vs. the BI + 40 mg/kg GA group (*n* = 6). (**H**) Representative images depicting H&E staining of liver and kidney tissues from the BI + 40 mg/kg GA + lentivirus-TCTN1 treatment group. (**I**) Representative images of H&E and Masson’s trichrome staining of carotid arteries. Histograms present the area ratios of intima to media as well as the proportions of smooth muscle fiber area (%). **, *p* < 0.01 compared to the sham group; ##, *p* < 0.01 in comparison with the BI group; ^, *p* < 0.05 relative to the BI + 40 mg/kg GA treatment group (*n* = 8). (**J**) The immunohistochemical analysis of ki67. The histogram displays the AOD of ki67. ***, *p* < 0.001 against the sham group; ##, *p* < 0.01 compared to the BI group; ^, *p* < 0.05 when contrasted with the BI + 40 mg/kg GA group (*n* = 8). (**K**) Immunofluorescence staining illustrates the colocalization of CD31 (red) and α-SMA (green). DAPI (blue) was used for nucleus staining. The lower section indicates the Pearson R values corresponding to the representative images. A histogram represents the Pearson R value for α-SMA-CD31 colocalization. **, *p* < 0.01 compared to the sham group; #, *p* < 0.05 against the BI group; ^, *p* < 0.05 relative to the BI + 40 mg/kg GA treatment group (*n* = 8).

To elucidate the molecular targets of GA (13:0), this study performed a comparative proteomic analysis using an in vitro PDGF-BB-mediated model, comparing samples with and without 1 μM GA (13:0) pretreatment. As shown in Figure 4A, a total of 216 proteins were found to be significantly differentially expressed, of which 108 proteins were up-regulated (Table S1) and 108 proteins were down-regulated (Table S2). The top 20 up-regulated and down-regulated proteins (Table 3) were labeled in a volcano plot and a clustering heatmap (Figure 4B). Among these proteins, we focused on TCTN1 due to its reported involvement in regulating cell proliferation and migration [40,41,42]. First, the effect of GA (13:0) on the expression of TCTN1 both in vitro and in vivo was assessed using Western blot and immunohistochemistry. As shown in Figure 4C,D, PDGF-BB significantly increased the transcription and protein levels of TCTN1. Pretreatment with 0.4, 0.8, and 1 μM GA (13:0) markedly inhibited PDGF-BB-induced increases in TCTN1 transcription and expression, while 0.2 μM GA (13:0) had no significant effect. Treatment with 1 μM GA (13:0) alone did not significantly alter TCTN1 transcription or expression in normal HA-VSMCs (Figure 4C,D). Consistent with this, in vivo results demonstrated that BI significantly induced an increase in TCTN1-positive expression, while i.p. injection of 40 mg/kg GA (13:0) markedly suppressed both the AOD value and positive expression of TCTN1 (Figure 4E). Lower doses (10 mg/kg and 20 mg/kg GA (13:0)) had no effect on BI-induced TCTN1 expression (Figure 4E). Second, to further validate that GA (13:0) inhibits VSMC proliferation and migration by reducing TCTN1 expression, we constructed the TCTN1 overexpression plasmid pcDNA3.1-EGFP-TCTN1 and the overexpression lentivirus Lenti-TCTN1 to observe the impacts of TCTN1 overexpression on VSMC proliferation, migration, and intimal thickening in the presence of GA (13:0) both in vitro and in vivo. Compared to the control pcDNA3.1 group, robust green fluorescence in the TCTN1 overexpression group confirmed successful overexpression in vitro (Figure 4F). TCTN1 overexpression significantly reversed the inhibitory effects of 1 μM GA (13:0) on PDGF-BB-induced cell proliferation and migration (Figure 4F). Western blot analysis in Figure 4G showed that BI dramatically increased TCTN1 protein levels in rat carotid arteries, and 40 mg/kg GA (13:0) significantly attenuated this increase, consistent with the immunohistochemistry results in Figure 4E. Lentiviral overexpression of TCTN1 significantly elevated TCTN1 protein levels under GA (13:0) treatment, confirming successful in vivo overexpression (Figure 4G). HE staining revealed no significant effect of TCTN1 overexpression on liver or kidney structure (Figure 4H). However, HE and Masson staining of carotid arteries in Figure 4I demonstrated that TCTN1 overexpression significantly reversed the inhibitory effects of 40 mg/kg GA (13:0) on BI-mediated intimal thickening and increased smooth muscle fiber, manifested as an increase in the intima/media area ratio (0.33 ± 0.1 vs. 0.48 ± 0.09, *p* < 0.05) and the proportion of smooth muscle fiber area (31.4 ± 6.6 vs. 44.3 ± 8.7, *p* < 0.05), respectively. Furthermore, overexpression of TCTN1 significantly reversed the GA (13:0)-induced reduction in ki67 expression (increased AOD value, *p* < 0.05; Figure 4J) and the decrease in CD31-α-SMA co-localization (increased Pearson R value, *p* < 0.05; Figure 4K).

### 3.4. GA (13:0) Regulates Cell Cycle Progression During VSMC Proliferation Through TCTN1

**Figure 5:**
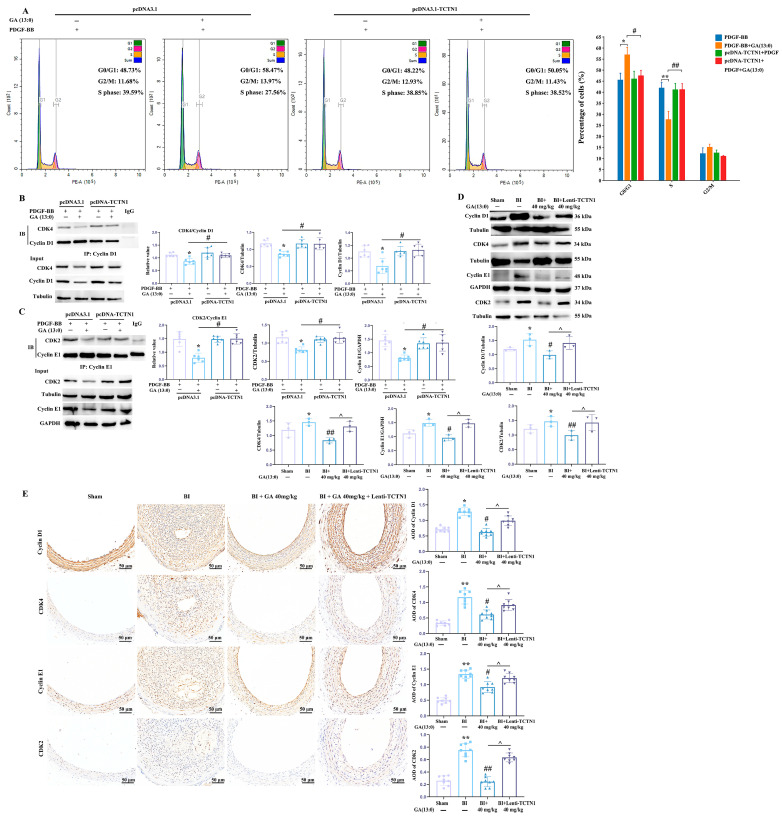
TCTN1 overexpression reverses the GA-induced G0/G1 phase arrest, as well as the inhibition of the expression and binding of cell cycle-related proteins. The treatment methods for cells and rats are the same as those in Figure 4. (**A**) Cell cycle progression was assessed using flow cytometry with PI staining. The histogram illustrates the quantification of cell cycle phases among various groups. *, *p* < 0.05 and **, *p* < 0.01 vs. the PDGF-BB + pcDNA3.1-EGFP group; #, *p* < 0.05 and ##, *p* < 0.01 vs. the PDGF-BB + 1 μM GA + pcDNA3.1-EGFP group (*n* = 6). (**B**) The expression and interaction of cyclin D1 and CDK4 were evaluated through Western blotting and Co-IP. Cyclin D1 was isolated from cell lysates using specific antibodies. The histograms represent the ratios of CDK4 to cyclin D1, CDK4 to tubulin, and cyclin D1 to tubulin. *, *p* < 0.05 vs. the PDGF-BB + pcDNA3.1-EGFP group; #, *p* < 0.05 vs. the PDGF-BB + 1 μM GA + pcDNA3.1-EGFP group (*n* = 6). (**C**) Western blot and Co-IP analyzed the expression and binding of cyclin E1 and CDK2. Cyclin E1 was immunoprecipitated using specific antibodies. Histograms show the ratios of CDK2 to cyclin E1, CDK2 to tubulin, and cyclin E1 to GAPDH. *, *p* < 0.05 vs. the PDGF-BB + pcDNA3.1-EGFP group; #, *p* < 0.05 vs. the PDGF-BB + 1 μM GA + pcDNA3.1-EGFP group (*n* = 6). (**D**) The expression levels of cyclin D1, CDK4, cyclin E1, and CDK2 in rat carotid arteries were assessed via Western blot analysis. The histograms present the ratios of cyclin D1 to tubulin, CDK4 to tubulin, CDK2 to tubulin, and cyclin E1 to GAPDH. *, *p* < 0.05 compared to the sham group; #, *p* < 0.05 and ##, *p* < 0.01 in comparison with the BI group; ^, *p* < 0.05 relative to the BI + 40 mg/kg GA (*n* = 3). (**E**) The immunohistochemistry results illustrated the presence of cyclin D1, CDK4, cyclin E1, and CDK2 in the carotid arteries of the rat BI model. The histograms depict the AOD for the target proteins. *, *p* < 0.05 and **, *p* < 0.01 compared to the sham group; #, *p* < 0.05 and ##, *p* < 0.01 vs. the BI group; ^, *p* < 0.05 when compared to the BI + 40 mg/kg GA (*n* = 8).

To further elucidate whether GA (13:0) regulates cell cycle progression and thereby controls VSMC proliferation through TCTN1, the effects of GA (13:0) on the cell cycle was examined in PDGF-BB-induced HA-VSMCs following TCTN1 overexpression. As shown in Figure 5A, compared to the pcDNA3.1 group, overexpression of TCTN1 significantly reversed the GA (13:0)-mediated increase in the proportion of cells in G0/G1 phase (from 57.06 ± 2.3% to 47.57 ± 2.48%; *p* < 0.05) and decreased in the proportion of cells in S phase (from 27.72 ± 3.74% to 41.31 ± 2.33%; *p* < 0.01). TCTN1 overexpression had no significant effect on the proportion of cells in G2/M phase. In addition, the effect of TCTN1 overexpression on GA (13:0)-mediated changes in cyclin expression and binding was assessed in the VSMC proliferation model. Results in Figure 5B indicate that TCTN1 overexpression dramatically reversed the decline in cyclin D1 and CDK4 expression, as well as the reduction in cyclin D1-CDK4 binding induced by 1 μM GA (13:0). Similarly, Figure 5C showed that TCTN1 overexpression significantly reversed the GA (13:0)-induced decrease in cyclin E1 and CDK2 expression as well as cyclin E1-CDK2 binding. The Western blot results in vivo demonstrated that i.p. injection of 40 mg/kg GA (13:0) significantly suppressed the BI-induced upregulation of cell cycle proteins (cyclin D1, CDK4, cyclin E1, and CDK2), corroborating the immunohistochemistry results in Figure 2D (Figure 5D). Overexpression of TCTN1 reversed the decrease in these cell cycle proteins induced by GA (13:0) treatment and markedly increased their levels (Figure 5D). Furthermore, as shown in Figure 5E, TCTN1 overexpression significantly increased the AOD values and positive expressions of cyclin D1, CDK4, cyclin E1, and CDK2, counteracting the reduction in these proteins induced by GA (13:0), consistent with the results in Figure 5D.

### 3.5. GA (13:0) Regulates the Vimentin and Actin Cytoskeletons Through TCTN1

**Figure 6:**
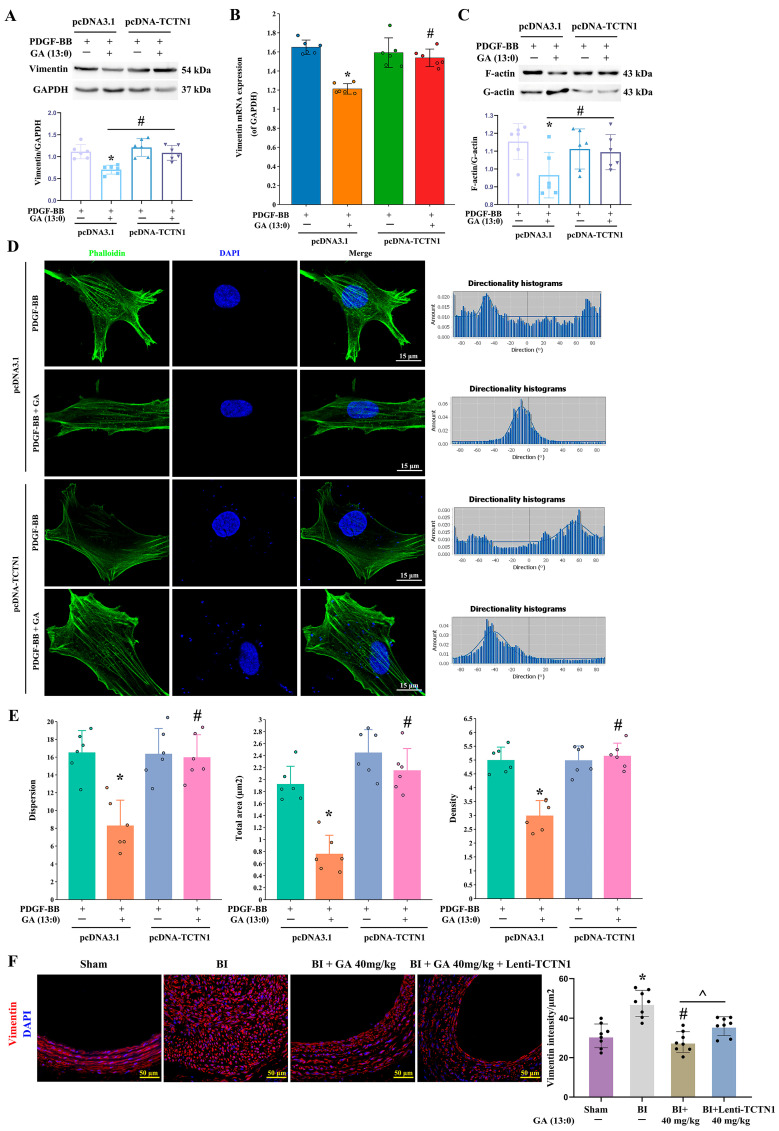
TCTN1 overexpression reverses the GA-induced inhibitory effects on vimentin expression and F-actin polymerization. The treatment methods for cells and rats are the same as those in Figure 4. (**A**) Western blot analysis of vimentin. Histogram shows the ratio of vimentin to GAPDH. *, *p* < 0.05 vs. the PDGF-BB + pcDNA3.1-EGFP group; #, *p* < 0.05 vs. the PDGF-BB + 1 μM GA + pcDNA3.1-EGFP group (*n* = 6). (**B**) qRT-PCR analysis of vimentin transcription. Histogram shows the ratio of vimentin mRNA to GAPDH mRNA. *, *p* < 0.05 vs. the pcDNA3.1-EGFP group; #, *p* < 0.05 vs. the PDGF-BB + 1 μM GA + pcDNA3.1-EGFP group (*n* = 6). (**C**) The ration of F-action to G-actin analyzed by G-actin/F-actin In vivo Assay Kit and Western blot analysis. Histogram shows the ratio of F-actin/G-actin. *, *p* < 0.05 vs. the PDGF-BB + pcDNA3.1-EGFP group; #, *p* < 0.05 vs. the PDGF-BB + 1 μM GA + pcDNA3.1-EGFP group (*n* = 6). (**D**) F-actin filaments were visualized using phalloidin (green), while nuclei were marked with DAPI (blue). The right panels show F-actin fiber directionality histogram, showing the directions and the distribution ratios of each direction. (**E**) Quantitatively analysis of the dispersion, total area and density of F-actin fibers in (**D**) by Image-Pro Plus software. *, *p* < 0.05 vs. the PDGF-BB + pcDNA3.1-EGFP group; #, *p* < 0.05 vs. the PDGF-BB + 1 μM GA + pcDNA3.1-EGFP group (*n* = 6). (**F**) The immunofluorescence staining for vimentin (red) also included DAPI staining for nuclei (blue). The histogram illustrates the vimentin intensity per μm^2^. *, *p* < 0.05 in comparison to the sham group; #, *p* < 0.05 relative to the BI group; ^, *p* < 0.05 vs. the BI + 40 mg/kg GA (*n* = 8).

To further investigate whether GA (13:0) regulates the cytoskeleton via TCTN1, thereby affecting VSMCs migration, we overexpressed TCTN1 in both in vivo and in vitro models to observe its ability to counteract the effects of GA (13:0). As shown in Figure 6A,B, TCTN1 overexpression significantly increased vimentin protein and mRNA levels in PDGF-BB-stimulated VSMCs treated with 1 μM GA (13:0), reversing the GA (13:0)-induced suppression of vimentin transcription and expression. Moreover, overexpression of TCTN1 significantly elevated the level of F-actin/G-actin ratio, counteracting the reduction in F-actin/G-actin ratio mediated by 1 μM GA (13:0) (Figure 6C). Figure 6D illustrated that TCTN1 overexpression promoted the multi-directional distribution of F-actin and attenuated the unidirectional F-actin pattern induced by 1 μM GA (13:0). TCTN1 overexpression also increased F-actin dispersion, total area, and fiber density, significantly mitigating the decreases caused by 1 μM GA (13:0) (Figure 6E). Vimentin immunofluorescence in the in vivo BI model revealed that overexpression of TCTN1 reversed the decrease in vimentin fluorescence intensity induced by 40 mg/kg GA (13: 0) treatment, significantly enhancing vimentin intensity in rat carotid arteries (Figure 6F).

Additionally, we analyzed the impact of GA (13:0) on the interaction between TCTN1 and cytoskeletal proteins. As no prior studies have demonstrated direct binding between TCTN1 and cytoskeletal proteins, we predicted potential TCTN1 interactions with actin and vimentin using GRAMM docking (https://gramm.compbio.ku.edu/gramm (accessed on 11 October 2024)). As shown in Figure S1A, the calculated binding energies (ΔG) were −10.1 kcal/mol for TCTN1-actin and −34.8 kcal/mol for TCTN1-vimentin. Both negative ΔG values indicate spontaneous binding potential between TCTN1 and these cytoskeletal scaffolds. In the in vitro PDGF-BB-mediated model, the Pearson R values for TCTN1 co-localization with actin and vimentin were significantly increased, and this effect was inhibited by treatment with 1 μM GA (13:0) (Figure S1B). Treatment with 1 μM GA (13:0) alone did not significantly affect TCTN1 co-localization with actin or vimentin in normal HA-VSMCs (Figure S1B). Consistent with the in vitro findings, in vivo results demonstrated that BI significantly enhanced the Pearson R values and co-localization of TCTN1 with both actin and vimentin in the vascular neointima, which was dramatically inhibited by i.p. injection of 20 or 40 mg/kg GA (13:0) (Figure S1C,D). Additionally, 10 mg/kg GA (13:0) significantly reduced the Pearson R value and co-localization of TCTN1-vimentin in the BI model (Figure S1D).

### 3.6. The Transcription Factor CTCF Regulates the Transcription and Expression of TCTN1

**Figure 7:**
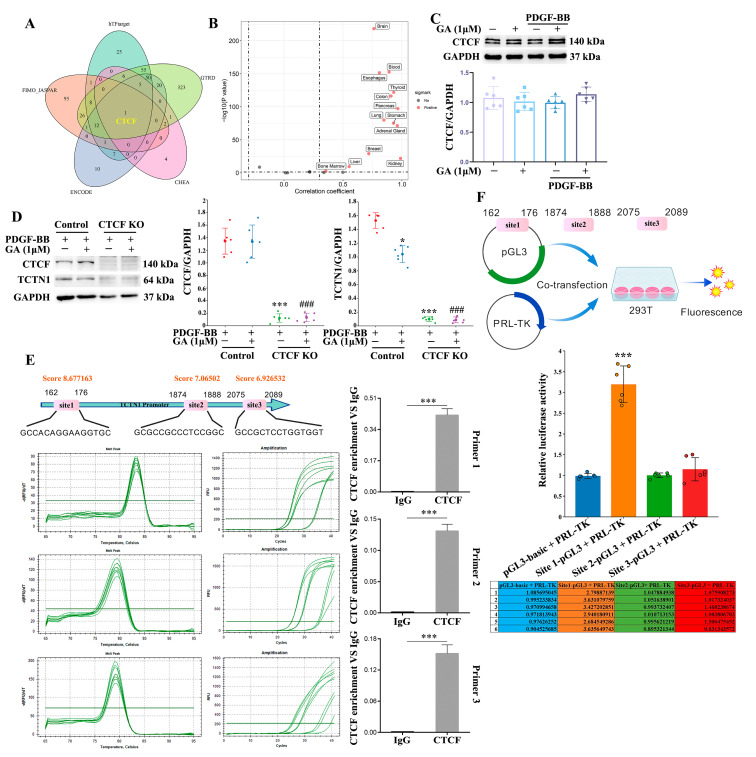
The transcription factor CTCF regulates TCTN1 gene expression by binding to site 1 (162–176) of the TCTN1 promoter. The HA-VSMC wild type and CTCF KO cells were pretreated with 1 μM GA (13:0) and then treated with 10 ng/mL PDGF-BB. (**A**) Screen for transcription factors CTCF that may regulate the expression of the target TCTN1 from the databases (hTFtarget, GTRD, CHEA, ENCODE, and FIMO_JASPAR). (**B**) Pan-tissue correlation analysis of CTCF regulating TCTN1 expression. Red dots indicate tissues where there is a correlation between CTCF and TCTN1 expression. (**C**) Analysis of CTCF via Western blot. The histogram illustrates the CTCF to GAPDH ratio. In PDGF-BB-induced HA-VSMCs, treatment with GA (13:0) did not have a significant impact on CTCF expression (*n* = 6). (**D**) Western blot analysis for CTCF and TCTN1 was performed in both wild type and CTCF KO HA-VSMCs. The histograms depict the ratios of CTCF and TCTN1 to GAPDH. *, *p* < 0.05 and ***, *p* < 0.001 compared to PDGF-BB-treated HA-VSMCs; ###, *p* < 0.001 in comparison with HA-VSMCs subjected to both PDGF-BB and 1 μM GA (*n* = 6). (**E**) JASPAR predicted the binding sites of CTCF on the TCTN1 promoter and verifies the binding of CTCF to these sites through ChIP-qPCR. The schematic diagram shows three binding sites with relatively high scores and the corresponding sequences. DNA was enriched by CTCF antibody or IgG (negative control). qPCR was performed using primers corresponding to the three binding sites. The left and middle panels, respectively, show the melt peak and amplification curves of qPCR. Histograms show the ratios of CTCF enrichment to IgG. ***, *p* < 0.001 vs. the IgG group (*n* = 3). (**F**) A Dual-Luciferase reporter assay was employed to measure the transcriptional activities of the above three binding sites. The schematic diagram shows the co-transfection of HEK293T cells with the recombinant pGL3 plasmid containing three sites and the pRL-TK plasmid. Histogram shows the relative luciferase activity. The table shows the specific values of the relative fluorescence activity of each group. ***, *p* < 0.001 vs. the pGL3-basic + pRL-TK group (*n* = 6).

To identify the transcription factor regulating TCTN1 expression, we used the TF-Target Finder tool—integrating data from multiple databases including hTFtarget, GTRD, CHEA, ENCODE, and FIMO_JASPAR—which revealed CTCF as a potential transcriptional regulator of TCTN1. Moreover, the TF-Target Finder analysis also indicated that CTCF could regulate TCTN1 transcription across a broad range of tissues, including the brain, lung, and stomach (Figure 7A,B). Next, we investigated whether CTCF regulates TCTN1 transcription in the PDGF-BB-mediated VSMC proliferation and migration model. As shown in Figure 7C, neither PDGF-BB treatment alone nor co-treatment with 1 μM GA (13:0) significantly affect CTCF protein levels in normal HA-VSMCs. Treatment with 1 μM GA (13:0) alone also did not significantly altered CTCF levels. By knocking out CTCF in HA-VSMCs using CRISPR/Cas9, CTCF protein band was significantly reduced compared to pre-knockout levels (*p* < 0.001), confirming successful CTCF knockout (Figure 7D). Notably, CTCF knockout significantly decreased TCTN1 expression in PDGF-BB-treated cells (*p* < 0.001). Furthermore, TCTN1 expression in the PDGF-BB + 1 μM GA (13:0) group was further decreased following CTCF knockout (*p* < 0.001) (Figure 7D). In order to identify CTCF binding sites within the TCTN1 promoter, we predicted three high-scoring sites using JASPAR: 162–176 (score 8.677163), 1874–1888 (score 7.06502), and 2075–2089 (score 6.926532) (Figure 7E). ChIP-qPCR confirmed CTCF binding to all three predicted sites (Figure 7E). The melt peaks and amplification curves from qPCR indicated reliable and specific data (Figure 7E). Importantly, enrichment of the TCTN1 promoter region was significantly higher in samples immunoprecipitated with CTCF antibody compared to the IgG control antibody. To assess the functional role of these sites in regulating TCTN1 transcription, we performed Dual-Luciferase reporter assays. As shown in Figure 7F, transfection with Site1-pGL3 significantly increased relative luciferase activity compared to the pGL3-basic vector control (*p* < 0.001). In contrast, no significant changes were observed in the Site2-pGL3 and Site3-pGL3 transfection groups.

## 4. Discussion

Vascular restenosis severely compromises the long-term efficacy and prognosis of interventional treatment. Neointima formation mediated by VSMC phenotypic transformation is a key contributor to vascular restenosis. While clinically used drugs for preventing vascular restenosis exist [9,10], their limitations necessitate the development of more effective alternatives. GAs, unique secondary metabolites of *Ginkgo biloba* found in leaves and seeds (highest in seed coat) [24], exhibit significant biological activities—including anti-inflammatory, antibacterial, antiviral, and insecticidal properties—despite safety concerns, highlighting their research value and potential as lead compounds. It has been reported that GA significantly inhibits inflammation and oxidative stress in ox-LDL-stimulated HUVECs, improves vascular intimal function, and shows potential application value in atherosclerosis treatment [24]. However, its role in preventing vascular restenosis was previously unknown. Among the six naturally occurring GA structures, C13:0, C15:1, and C17:1 are more abundant in GA preparations and extensively studied. GA (13:0) was selected for this investigation due to the absence of double bonds in its hydrocarbon group, potentially reducing allergenic risk [43]. This study reveals for the first time that i.p. injection of GA (13:0) can significantly suppress the proliferation and migration of VSMCs as well as neointima formation, with no observed structural damage to the liver or kidneys.

A previous study reported that GBE inhibits the proliferation of hepatic stellate cells via cell cycle arrest [44]. Consistent with this, our findings demonstrate that GA (13:0) suppresses VSMC proliferation through interfering with cell cycle regulatory mechanisms. In the PDGF-BB-induced VSMC proliferation model, GA (13:0) at concentrations of 0.8 and 1 μM significantly blocked the G1/S transition and induced G0/G1 arrest. This arrest was associated with the downregulation of core cell cycle regulators—cyclin D1, cyclin E1, CDK2, and CDK4—and the disruption of cyclin D1–CDK4 and cyclin E1–CDK2 complex formation. It has been reported that the G1/S transition is regulated by cyclin D–CDK4 activity in the early G1 phase and by cyclin E–CDK2 activity in the late G1 phase [45]. Thus, our findings suggest that GA (13:0) acts on both key checkpoints in the G1 phase to halt cell cycle progression. Notably, in vivo experiments revealed differential sensitivity among cell cycle proteins to GA (13:0): CDK4 expression was significantly suppressed at 10 mg/kg GA (13:0), whereas cyclin D1 and cyclin E1 levels were reduced only at the higher dose of 40 mg/kg. Similarly, in vitro analyses showed that the cyclin E1–CDK2 complex was disrupted at 0.8 μM GA (13:0), while inhibition of the cyclin D1–CDK4 complex required 1 μM GA (13:0). This dose-dependent pattern suggests that GA (13:0) modulates cell cycle progression through multiple targets and at multiple levels. It appears that GA (13:0) preferentially targets CDK4 in early G1 and the cyclin E1–CDK2 complex in late G1, with higher doses further suppressing cyclin expression and impairing cyclin D1–CDK4 assembly. In contrast to prior reports [44], this study systematically elucidates the role of GA (13:0) in cell cycle regulation within a vascular restenosis model. Vimentin and actin cytoskeletons are closely related to VSMC migration [33,41]. Here, we report for the first time that GA (13:0) interferes with cytoskeletal dynamics, thereby suppressing VSMC migration. GA (13:0) not only reduced vimentin expression but also promoted F-actin depolymerization and induced a reorganization of its spatial architecture, shifting the cytoskeleton from a multidirectional to a unidirectional alignment. These findings provide novel insight into the pharmacological functions of GA compounds from the perspective of cytoskeletal dynamics. While previous studies [46] have focused on signaling pathways regulating cell invasion by GA, our work highlights an alternative mechanism—cytoskeletal remodeling—as a pathway through which GA (13:0) impedes cell migration.

To further identify the molecular target of GA (13:0), we performed comparative proteomics and identified TCTN1 as a key downregulated protein. TCTN1 has been implicated in cell proliferation and migration [40,41,42] and acts as a novel regulator of the Hedgehog signaling pathway, playing important roles in ciliogenesis, cell cycle control, and signal transduction [47,48,49]. Studies have shown that TCTN1 is aberrantly expressed in several solid tumors, and its knockdown can induce S-phase arrest by regulating CDKs and cyclins, thereby suppressing tumor cell proliferation and migration while promoting apoptosis [40,41,45,50,51]. However, the role of TCTN1 in VSMC phenotypic switching and vascular restenosis remains unexplored. Here, we demonstrate for the first time that TCTN1 expression is upregulated in a model of intimal hyperplasia and is significantly reversed by GA (13:0). Moreover, overexpression of TCTN1 counteracted the inhibitory effects of GA (13:0) on VSMC proliferation, migration, and neointima formation. These results indicate that the core mechanism by which GA (13:0) exerts its effects involves downregulating TCTN1, thereby suppressing the expression and complex formation of cell cycle-related proteins, as well as inhibiting vimentin and actin cytoskeletal reorganization—collectively hindering cell cycle progression and migration. Notably, we observed that GA (13:0)-induced G0/G1 arrest differs from the S-phase or G2/M arrest reported in cancer studies following TCTN1 knockdown [45,50], suggesting cell type-specific regulatory roles of TCTN1. In addition, although TCTN1 acts as a protein scaffold that promotes tumor cell migration [41], its relationship with canonical cytoskeletal proteins has remained unclear. Our study provides preliminary evidence of a close interaction between TCTN1 and the vimentin/actin cytoskeleton and shows that GA (13:0) modulates this interaction. These findings offer new insights into the mechanism by which TCTN1 regulates cell migration and the pharmacological action of GA (13:0). Further studies are warranted to clarify the nature of TCTN1–cytoskeleton interactions and how GA (13:0) precisely targets this process.

While the transcription factor AP2α has been reported to regulate TCTN1 expression and promote proliferation, migration, and invasion in oral squamous cell carcinoma (OSCC) [52], our study identifies CTCF—rather than AP2α—as a key transcriptional regulator of TCTN1. Using promoter-binding assays, we confirmed that CTCF binds to a specific region (positions 162–176) of the TCTN1 promoter and functionally regulates its transcription. Although GA (13:0) did not alter CTCF expression levels, it likely suppresses TCTN1 transcription by interfering with CTCF’s binding activity at the promoter. Further experiments are needed to fully elucidate the precise molecular mechanism through which GA (13:0) modulates CTCF-dependent transcriptional regulation of TCTN1.

In conclusion, the main conclusions of this study are as follows: (1) GA (13:0) significantly inhibits VSMC proliferation and migration as well as vascular restenosis by reducing TCTN1 expression; (2) GA (13:0) promotes G0/G1 arrest by inhibiting the expression and binding of cell cycle-related proteins via TCTN1; (3) GA (13:0) regulates VSMC migration by modulating vimentin expression and actin rearrangement through TCTN1; (4) GA (13:0) regulates TCTN1 transcription and expression through the transcription factor CTCF. This study reveals that GA (13:0) modulates TCTN1 expression via CTCF, thereby regulating cell cycle progression and cytoskeleton rearrangement to inhibit VSMC proliferation, migration, and vascular restenosis. This provides a basis for developing GA as a potential therapeutic compound for vascular restenosis.

## Figures and Tables

**Table 1:** The primers for qRT-PCR.

Primer Name	Sequence
Vimentin-F	5′-GACGCCATCAACACCGAGTT-3′
Vimentin-R	5′-CTTTGTCGTTGGTTAGCTGGT-3′
GAPDH-F	5′-ACAACTCTCTCAAGATTGTCAGC-3′
GAPDH-R	5′-ACTTTGTGAAGCTCATTTCCTGG-3′
TCTN1-F	5′-TGTTCAGTCCATCGTCATTCAG-3′
TCTN1-R	5′-GCAAAGGCTAAAGTGTCCAGC-3′

**Table 2:** The primers of ChIP-qPCR.

Primer Name	Sequence
TCTN1-Primer1-F	5′-AAGCAATCCTTTGCAGAAAAA-3′
TCTN1-Primer1-R	5′-AAGCAATCCTTTGCAGAAAAA-3′
TCTN1-Primer2-F	5′-GCTTCACACCCGCTCACTA-3′
TCTN1-Primer2-R	5′-AAGCTGCCGGAGACTGACT-3′
TCTN1-Primer3-F	5′-CTCCTGGTGGTGCTCCTG-3′
TCTN1-Primer3-R	5′-GGTGGCCAGGGCTGCCTCG-3′

**Table 3:** TOP 20 up- and down-regulated proteins.

Accession No.	Symbol	Protein Name
TOP 20 up-regulated proteins		
Q05932	FPGS	Folylpolyglutamate synthase, mitochondrial
Q8WUY3	PRUNE2	Protein prune homolog 2
P49810	PSEN2	Presenilin-2
Q8IY26	PLPP6	Polyisoprenoid diphosphate/phosphate phosphohydrolase PLPP6
Q9BYR8	KRTAP3-1	Keratin-associated protein 3-1
Q9BYR6	KRTAP3-3	Keratin-associated protein 3-3
Q9BV73	CEP250	Centrosome-associated protein CEP250
Q00266	MAT1A	S-adenosylmethionine synthase isoform type-1
Q99633	PRPF18	Pre-mRNA-splicing factor 18
Q9Y4K1	CRYBG1	Beta/gamma crystallin domain-containing protein 1
P25311	AZGP1	Zinc-alpha-2-glycoprotein
P05062	ALDOB	Fructose-bisphosphate aldolase B
O75319	DUSP11	RNA/RNP complex-1-interacting phosphatase
Q6ZV89	SH2D5	SH2 domain-containing protein 5
P11831	SRF	Serum response factor
O75140	DEPDC5	GATOR1 complex protein DEPDC5
Q15493	RGN	Regucalcin
Q9HCG7	GBA2	Non-lysosomal glucosylceramidase
Q5TEA3	DNAAF9	Dynein axonemal assembly factor 9
Q6Q0C0	TRAF7	E3 ubiquitin-protein ligase TRAF7
TOP 20 down-regulated proteins		
Q8IV20	LACC1	Purine nucleoside phosphorylase LACC1
P43251	BTD	Biotinidase
Q96C10	DHX58	ATP-dependent RNA helicase DHX58
Q15910	EZH2	Histone-lysine N-methyltransferase EZH2
Q8NDN9	RCBTB1	RCC1 and BTB domain-containing protein 1
Q9NYV6	RRN3	RNA polymerase I-specific transcription initiation factor RRN3
O15360	FANCA	Fanconi anemia group A protein
Q9UPM9	B9D1	B9 domain-containing protein 1
A7KAX9	ARHGAP32	Rho GTPase-activating protein 32
Q9Y421	FAM32A	Protein FAM32A
Q6PJ69	TRIM65	E3 ubiquitin-protein ligase TRIM65
Q8NEZ4	KMT2C	Histone-lysine N-methyltransferase 2C
Q8NDZ2	SIMC1	SUMO-interacting motif-containing protein 1
Q2M3G0	ABCB5	ATP-binding cassette sub-family B member 5
Q8N1A6	C4orf33	UPF0462 protein C4orf33
Q9H4B6	SAV1	Protein salvador homolog 1
Q7RTP0	NIPA1	Magnesium transporter NIPA1
Q2MV58	TCTN1	Tectonic-1
O43422	THAP12	52 kDa repressor of the inhibitor of the protein kinase
Q71F56	MED13L	Mediator of RNA polymerase II transcription subunit 13-like

## Data Availability

The data used to support the findings of this study are available from the corresponding authors upon request.

## Supplementary Materials

The following supporting information can be downloaded at: https://www.mdpi.com/article/10.3390/cells14231922/s1, Figure S1: GA (13:0) significantly inhibits the co-localization of TCTN1 with actin and vimentin respectively in the PDGF-BB-induced HA-VSMCs in vitro and the BI rats in vivo. (A) The potential TCTN1 interactions with actin and vimentin were predicted using GRAMM docking. The binding energy (ΔG) for TCTN1-actin was −10.1 kcal/mol, and for TCTN1-vimentin was −34.8 kcal/mol. Both negative ΔG values indicate spontaneous binding potential between TCTN1 and these cytoskeletal scaffolds. (B–D) Immunofluorescence staining of the colocalization of TCTN1 (green) with actin (red) or vimentin (red). Nuclei were stained with DAPI (blue). The lower panels in (C) or (D) show the Pearson R values for the representative images. Histograms show the Pearson R values of TCTN1-Actin or TCTN1-Vimentin colocalization. (B) *, *p* < 0.05 and **, *p* < 0.01 vs. the control group; #, *p* < 0.05 vs. the PDGF-BB-treated group (*n* = 6). (C,D) **, *p* < 0.01 vs. the sham group; #, *p* < 0.05 and ##, *p* < 0.01 vs. the BI group (*n* = 8); Table S1: Proteins significantly upregulated after GA (13:0) treatment; Table S2: Proteins significantly downregulated after GA (13:0) treatment.
