# Supplementary figures and images for: Ginkgolic Acid Inhibits VSMC Proliferation and Migration and Vascular Restenosis by Regulating Cell Cycle Progression and Cytoskeleton Rearrangement Through TCTN1
Source: Cells. 2025 Dec 3;14(23):1922. doi: 10.3390/cells14231922 (PMC12691368; doi:10.3390/cells14231922)

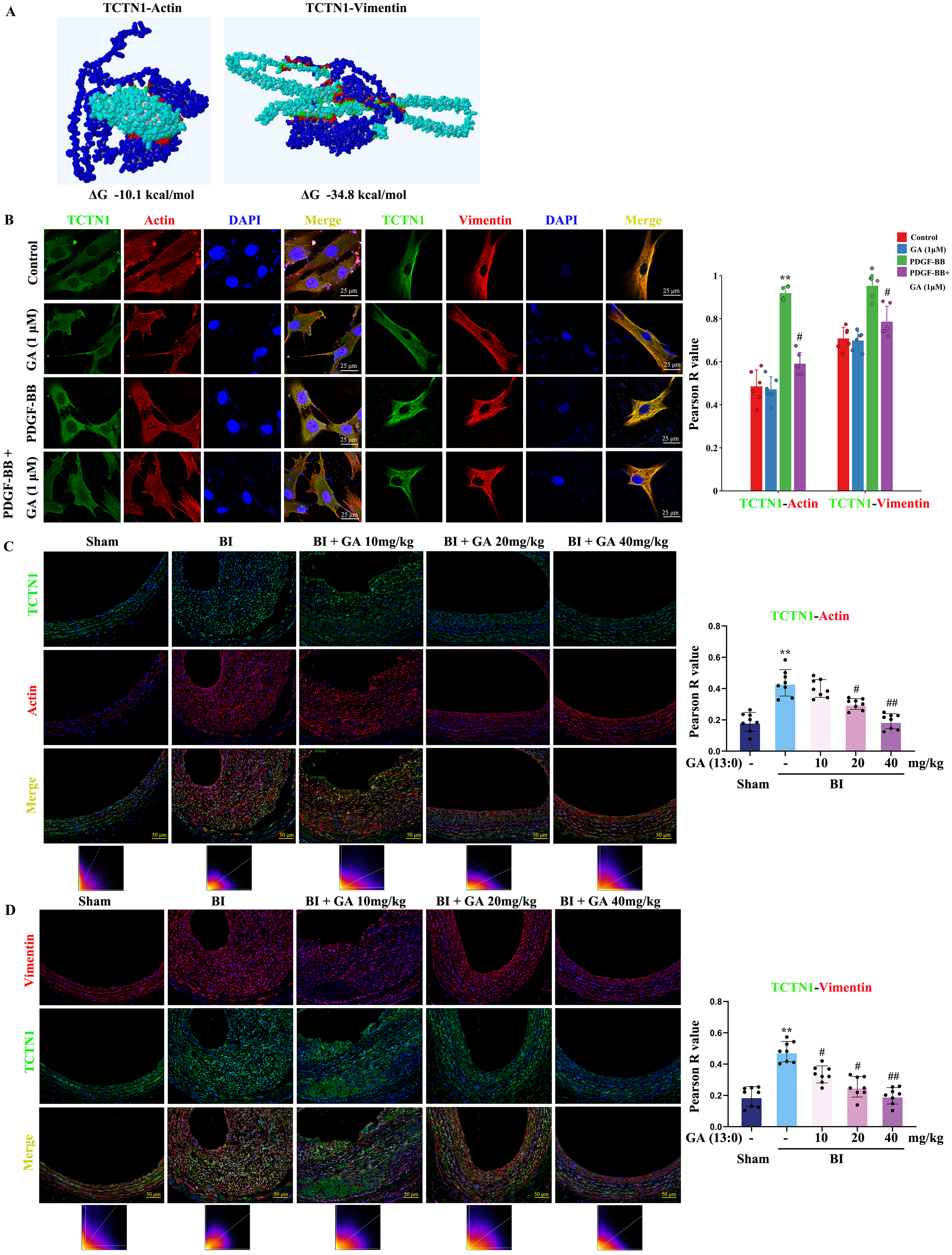

Supplement: Supplementary file 1 [file cells-14-01922-s001.zip › supplementary data/Fig. S1.tif]
